# Modeling the effect of bilateral sagittal split osteotomy on posterior, superior and medial space dimensions of the temporomandibular joint: a retrospective controlled cohort study

**DOI:** 10.1186/s12903-023-02959-3

**Published:** 2023-05-17

**Authors:** Linus Christian Hupp, Michael Verius, Annika Bertram, Andreas Kolk, Rüdiger Emshoff

**Affiliations:** 1 University Clinic of Oral and Maxillofacial Surgery, Medical University of Innsbruck, Anichstraße 35, 6020 Innsbruck, Austria; 2 University Clinic of Radiology, Medical University of Innsbruck, Innsbruck, Austria; 3 Department of Neuroradiology, University of Heidelberg, Heidelberg, Germany

**Keywords:** Orthognathic surgery, Bilateral sagittal split ramus osteotomy, Temporomandibular joint, Condylar displacement, Cone-beam computed tomography

## Abstract

**Background:**

To model the effect of isolated bilateral sagittal split osteotomy (BSSO) on changes in posterior (PSD), superior (SSD), and medial space dimensions (MSD) of the temporomandibular joint.

**Methods:**

Using a retrospective cohort study design, pre- and postoperative (immediately after surgery; 1 year follow-up) cone-beam computed tomography measurements of 36 patients who had undergone BSSO for mandibular advancement were compared with a control group of 25 subjects from whom a mandibular odontogenic cyst was removed under general anesthesia. Generalized estimation equation (GEE) models were used to examine the independent effects of study group, preoperative condylar position, and time points on PSD, SSD, and MSD adjusting for covariates (age, sex, and mandibular advancement).

**Results:**

No significant differences were found regarding changes in PSD (*p* = 0.144), SSD (*p* = 0.607), or MSD (*p* = 0.565) between the BSSO and control groups. However, the preoperative posterior condylar position showed significant effects on PSD (*p* < 0.001) and MSD (*p* = 0.043), while the preoperative central condylar position demonstrated a significant effect on PSD (*p* < 0.001).

**Conclusion:**

The data suggest that preoperative posterior condylar position is a significant effect modifier of PSD and MSD over time in this cohort.

## Background

Orthognathic surgery refers to the surgical correction of developmental growth abnormalities of the jaws and facial bones [1]. These oral and maxillofacial (OMF) surgery procedures, which are usually performed under general anesthesia (GA), have been described to induce postoperative condylar displacements of the temporomandibular joint (TMJ), thereby becoming a significant factor in the occurrence of early and long-term skeletal relapse in the surgical correction of skeletal deformities [2–5]. Cone-beam computed tomography (CBCT) has become a standard for the purposes of 3D planning and follow-up of bimaxillary orthognathic surgery procedures and for the performance of accurate and reliable measurements within the 3-dimensional bony TMJ complex [6, 7]. For this reason CBCT has been recommended for evaluating condylar displacement after orthognathic surgery [8].

The application of CBCT has been reported for determining changes in condylar position after bilateral sagittal split ramus osteotomy (BSSO) for mandibular advancement, without [9–14] or with [15–18] a LeFort I osteotomy. According to these studies, condylar displacement after mandibular advancement surgery is variable. Posterior [16], posteroinferior [14, 15], inferoanterior [17], and posterosuperior directions [9–13] have been reported for short-term (2–12 weeks) and anteroinferior [10], posteroinferior [14], posterosuperior [12, 15], and superomedial [18] for long-term (6–12 months) displacements.

Although the occurrence of postoperative condylar displacements following mandibular advancement surgery are acknowledged to be of multifactorial origin, surgical intervention factors such as surgical intervention type, fixation method, direction and magnitude of mandibular advancement, and time intervals of measurement are cited as the major influences in the literature [19]. However, several authors have supported the contention that skeletal or demographic aspects may contribute to the variability of postoperative condylar position changes. Numerous studies disclosed that condyles in Class II patients were located more anteriorly than in those with a Class I or III malocclusion [20], thereby emphasizing the pronounced tendency of relapse after postoperative posterior condylar displacement. Further, it has been shown that women, especially if they are young, are at higher risk of developing postoperative condylar resorption than men, thereby possibly influencing the extent of postoperative condylar displacements [21].

To the knowledge of the authors there are no CBCT studies available addressing the occurrence of condylar displacements following surgical corrections of skeletal deformities in a controlled cohort study. Furthermore, these studies fail to address the three-dimensional changes in TMJ joint spaces with a multivariate design, i.e., baseline clinical and CBCT parameters were not taken into consideration to model the respective changes for the posterior (PSD), superior (SSD), and medial space dimensions (MSD) separately. Therefore, the purpose of this controlled cohort study was to model the effect of isolated BSSO for mandibular advancement on changes in PSD, SSD, and MSD, including an operative control group of patients, in whom mandibular odontogenic cysts were removed under GA.

## Methods

### Study design, population, inclusion and exclusion criteria

To address the research purpose, the investigators designed and implemented a retrospective controlled cohort study. The study population, selected over a period of approximately 2 years, was composed of consecutive patients with an isolated skeletal Class II malocclusion and patients in whom odontogenic cysts were removed under general anesthesia. Patients in the study group underwent isolated BSSO in the Department of Oral and Maxillofacial Surgery at the University of Innsbruck. To be included in the study sample, patients had to be diagnosed with skeletal Class II malocclusion without an anterior open bite. Patients were excluded as study subjects if they presented with (1) a bad split during their surgical operation (2) radiographic signs of osteoarthrosis according to the diagnostic criteria for temporomandibular disorders (DC/TMD) (i.e., generalized sclerosis, erosion, subchondral cyst, osteophyte) [22], or (3) a deformity secondary to trauma, severe facial asymmetry, cleft lip and palate, or systemic disease. Standardized lateral cephalometric radiographs and CBCTs of the right and left TMJs were routinely obtained 1 week preoperatively (T0), 3 to 5 days postoperatively (T1), and 1 year postoperatively (T2) to assess short- and long-term adaptive changes in the condyle position. All BSSO procedures were performed by one oral and maxillofacial surgeon with more than 15 years of experience performing orthognathic surgeries.

Patients in the control group were referred to the Department of Oral and Maxillofacial Surgery for odontogenic cyst removal. Inclusion criteria for patients in the control group were the presence of an odontogenic cyst in the posterior region of the mandible warranting pre- (1 week preoperatively (T0)) and postoperative CBCT assessments (3 to 5 days postoperatively (T1) and 1 year postoperatively (T2)). All odontogenic cysts were surgically removed by simple enucleation through an intraoral approach. Those who presented with radiographic signs of osteoarthrosis according to the diagnostic criteria for TMD (DC/TMD) (i.e., generalized sclerosis, erosion, subchondral cyst, osteophyte) [22], deformity secondary to trauma, severe facial asymmetry, cleft lip and palate, or systemic disease were excluded. The subjects were informed about the study procedure and written informed consent was received. The study followed the Declaration of Helsinki regarding medical protocol and ethics and was given approval in accordance with the guidelines of the local ethical committee (IMU IRB, Ref: 415-E/2181).

### Variables

The primary study outcomes were PSD, SSD, and MSD assessed at follow-ups with CBCT. The predictor variables were study group, preoperative condylar position, and time points on PSD, SSD, and MSD (T0, T1, and T2). Covariates included age, sex, and amount of mandibular advancement.

### Surgical technique

BSSO was performed to advance the mandible applying the Obwegeser technique [23], with the modification of Dal Pont [24]. After advancement of the distal segment, removable self-retentive acrylic splints and intermaxillary fixation with stainless steel wires were used in all patients to secure the desired occlusion intraoperatively. The ascending ramus was not fixed intraoperatively, and the condylar position was determined as its most relaxed centric rotational position by manipulating the condyle to the most superior position, and repeatedly releasing the condyle until a stable and reproducible position was found. Rigid internal fixation of the bony segments was achieved with three bicortical screws (2.0 mm in diameter and 14 mm in length) at each osteotomy site, as described by Van Sickels et al. [25]. The proximal and distal segments were passively positioned to one another when placing the bicortical screws to prevent any rotation or torquing of the condyle within the glenoid fossa [26].

### Postsurgical protocol

Semirigid elastics were used on the second postoperative day to keep the mandible in the new proper occlusion; these were maintained for 2 weeks. Thereafter, guiding elastics were applied to ensure proper occlusion and to regain regular mouth opening. The occlusal acrylic splint was removed 1 month after surgery. Approximately 6- to 8 weeks after surgery orthodontic treatment was initiated.

### CBCT data acquisition

A CBCT machine (KaVo 3D eXam; KaVo Dental GmbH, Biberach, Germany) was used to evaluate the relation of the condyle to the fossa. All patients sat in an upright position with the teeth in centric occlusion. The patients' Frankfort horizontal (FH) plane was parallel to the floor. The scanning settings of the CBCT machine were as follows: 16- × 13-cm field of view, 90-kVp tube voltage, 8.0-mA tube current, and 24-s scan time. The CBCT data were reconstructed with 3-dimensional image dental software (OnDemand3D; E KaVo Dental GmbH, Biberach, Germany). Lateral cephalometric measurements were obtained using Dolphin 3D Imaging software (Dolphin Imaging and Management Solutions, Chatsworth, CA, USA). Patient’s sagittal jaw relationship was classified according to the A point-nasion-B-point (ANB) angle into skeletal maloccluion Class I (ANB ranging from 0° to 4°), Class II (ANB > 4°), or Class III (ANB < 0°)[27].

### Sagittal and coronal scan images

On the axial scan images, the slice with the greatest mediolateral dimension of the condyle was identified and used as the reference image in the left and right TMJ. The image of the sagittal and coronal plane was determined through the middle point of the condyle on the axial plane.

### Evaluation of joint spaces and condylar position

**Fig. 1:**
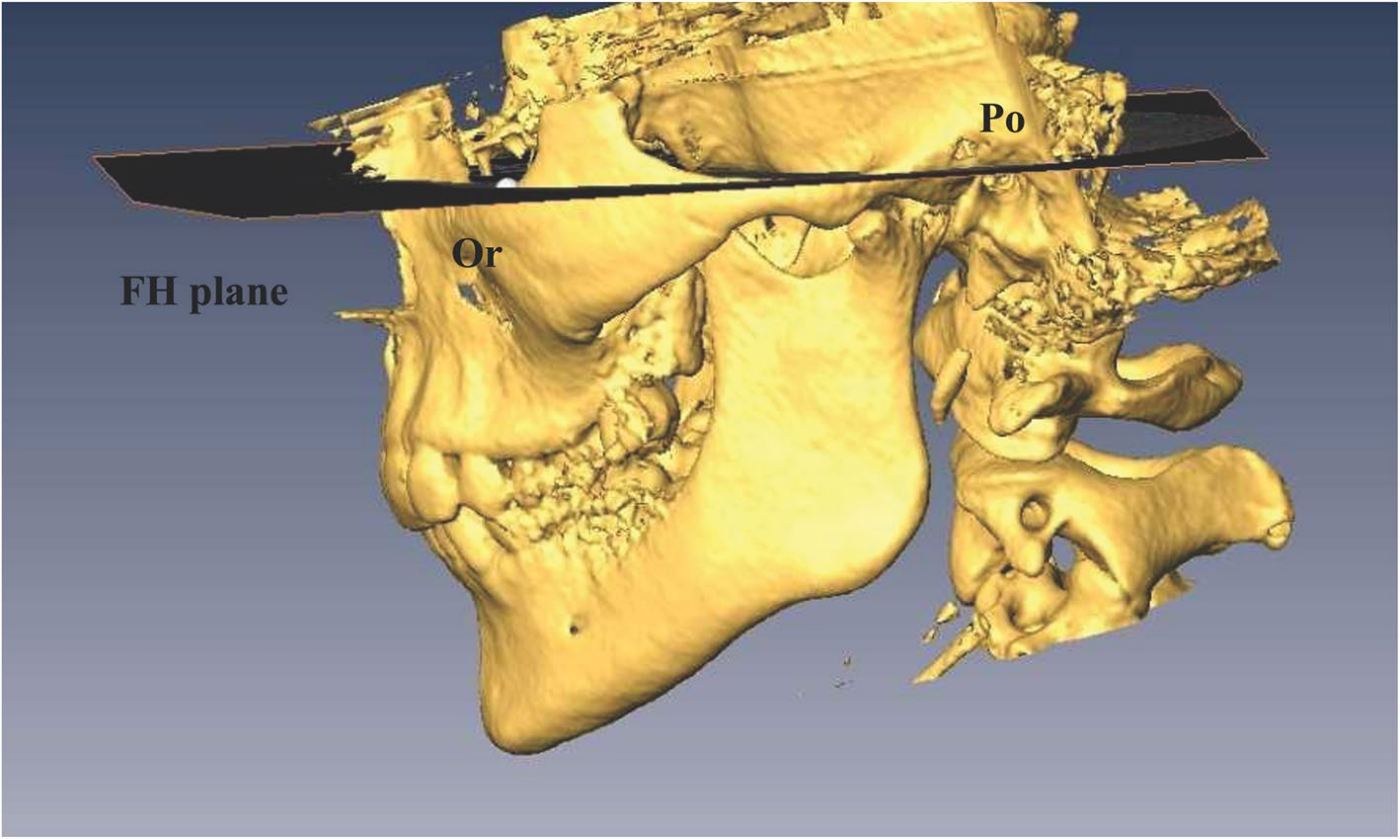
The FH plane was constructed by both sides of the Po and the right side of the Or. FH, Frankfort horizontal; Or, orbitale; Po, porion

**Fig. 2:**
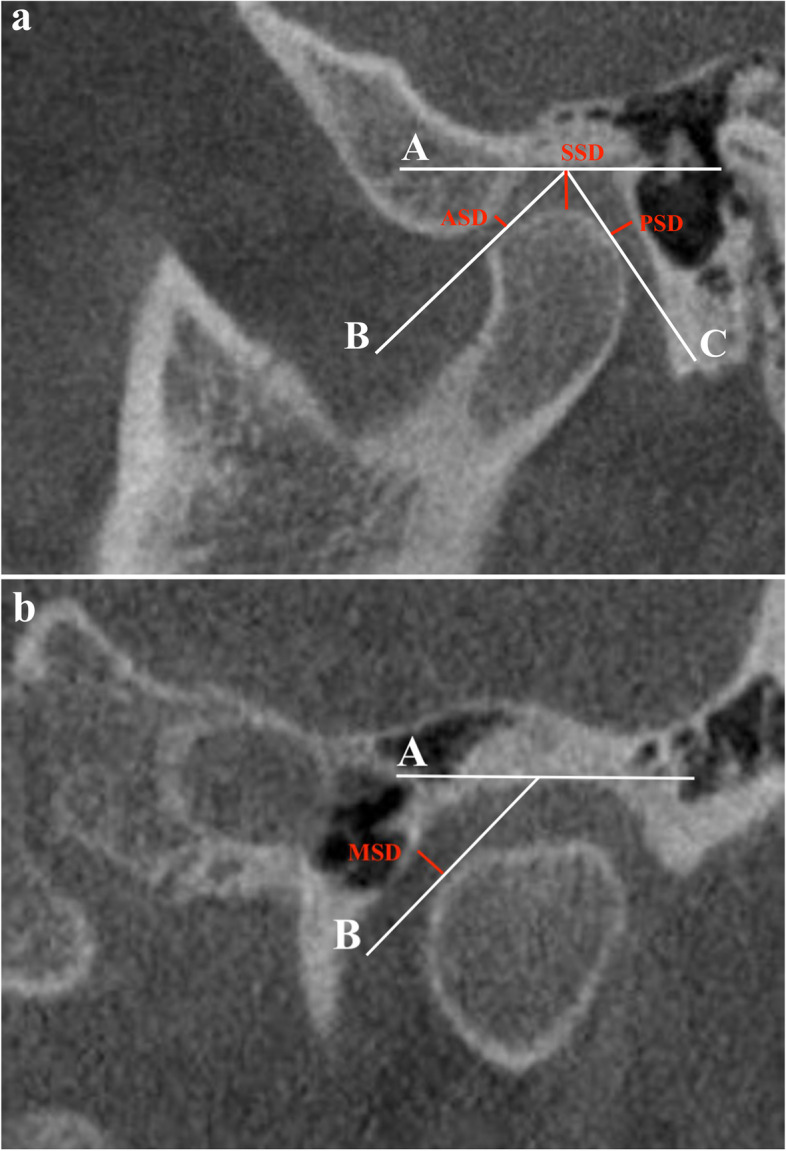
Assessment of the anterior (ASD), posterior (PSD), superior (SSD), and medial space dimensions (MSD). **a** Sagittal view with Line A drawn through the most superior aspect of the glenoid fossa parallel to the FH plane. The lines tangent to the most prominent anterior (Line B) and posterior (Line C) condylar surfaces were constructed from the most superior surface of the glenoid fossa. ASD and PSD were measured from the most prominent anterior and posterior points of the condyle to the glenoid fossa. SSD was the vertical distance from the most superior aspect of the glenoid fossa to the condyle. **b** Coronal view with Line A drawn through the most superior aspect of the glenoid fossa perpendicular to the FH plane. The line tangent to the most prominent medial condylar surface (Line B) was constructed from the most superior surface of the glenoid fossa. The MSD was measured from the most prominent medial point of the condyle to the glenoid fossa

The FH plane was constructed by the right and left sides of the porion and the right side of the infraorbitale (Fig. 1). Line A was drawn through the most superior surface of the glenoid fossa parallel to the FH plane. Anterior, superior, posterior, and medial spaces were measured by the method of Chae et al. [28] with 3 times enlargement for the multiplanar reconstruction image (Fig. 2). The anterior space dimension (ASD) and PSD were assessed and ln(PSD/ASD) was calculated by the method of Pullinger and Hollender [29] to determine the anteroposterior relation of the condyle to the fossa. A ln(PSD/ASD) > 0.25 indicated an anterior position, an ln(PSD/ASD) ≤ 0.25 and ≥ -0.25 a concentric, and a ln(PSD/ASD) < -0.25 a posterior position of the condyle in the glenoid fossa. Measurements were repeated 2 times and the mean value was used for analysis. One radiologist made the measurements using a digital ruler graded in mm.

For assessment of intraobserver reliability, linear dimensions of joint spaces in the CBCT images of 30 randomly selected cases were evaluated and measured by the investigator on two different days. For the CBCT measurements, the mean difference was 0.070 ± 0.21 mm; the intraclass correlation coefficient for intraobserver agreement accounted for 0.974.

### Statistics

The number of subjects for this study was set on the basis of a significance level of 0.05, a medium effect size of 0.24, and a power of 0.80, and a total of 6 variables were required for prediction modeling. As a result of the analysis using G*power 3.1 software, the minimum number of samples was 94, indicating that this study satisfies the appropriate number of samples.

Possible differences in baseline variables between the study groups were analyzed using independent sample t tests and chi-square analysis. Paired sample t test, independent sample t test, and chi-square analysis were applied to analyze joint space and condylar positional changes. Generalized estimation equation (GEE) models were used to examine the independent effects of study group, condylar position at baseline, and time on PSD, SSD, and MSD adjusting for covariates (age, sex, and amount of mandibular advancement). The significance of the two-way interaction of time and BSSO group (time*BSSO group) was tested using the GEE models to determine their synergistic effects on space dimensions. Statistical significance was set at *p* < 0.05. For the statistical analysis, the PASW 27.0 (SPSS Statistics, IBM, Chicago) package was used.

## Results

A total of 61 participants fulfilled the inclusion criteria. The BSSO sample was composed of 72 TMJs of 36 patients (28 females; 8 males; average age 30.5 ± 9.2 years) with a skeletal Class II malocclusion. In the control group of 50 TMJs of 25 subjects (8 females; 17 males; average age 37.7 ± 14.6 years), the skeletal relationship was Class I in 12, Class II in 3, and Class III in 10 subjects. For patients of the BSSO group, the advancement of the mandible at the B point ranged from 2 to 10 mm (mean, 6.1 mm ± 1.6 mm) (Table 1).

**Table 1:** Baseline sample characteristics of TMJs of the BSSO and control group

	**BSSO group (*n* = 72)**	**Control group (*n* = 50)**	**Total (*n* = 122)**	***P***
Age (years) (mean ± SD)	37.7 (14.6)	30.3 (6.2)	33.3 (12.2)	0.017^§^
Gender (n) (% female)	56 (77.8)	16 (32.0)	72 (59.0)	< 0.001^#^
Mandibular advancement (mm)	6.1 ± 1.6	0	3.6 ± 3.2	
Condylar position				
Anterior (%)	32 (44.4)	16 (32.0)	48 (39.3)	0.166
Concentric (%)	24 (33.3)	27 (54.0)	51 (41.8)	0.023^#^
Posterior (%)	16 (22.2)	7 (14.0)	23 (18.9)	0.253

*TMJ* Temporomandibular joint, *BSSO* Bilateral sagittal split osteotomy, *SD* Standard deviation, *mm* Millimeters, *n* Number of TMJs, *P* Probability of type I error

^*§*^ Significant with independent sample t-test

^*#*^ Significant with chi-square analysis

Analysis of the data showed the BSSO group to be characterized by a higher prevalence of females (77.8% vs. 32.0%) (*p* < 0.001) and a higher mean age (37.7 years vs. 30.3 years) (*p* = 0.017), whereas the control group was associated with a higher frequency of preoperative concentric condylar position (54.0% vs. 33.3%) (*p* = 0.023) (Table 1).

For the BSSO group, the PSD of the right (*p* < 0.001) and left TMJ sides (*p* < 0.001) decreased significantly over time; in addition, a significant decrease over time was observed for the SSD of the left TMJ side (*p* = 0.032) (Table 2). For the control group, the total SSD of both TMJ sides showed a significant decrease over time (*p* = 0.023) (Table 3). The overall changes in posterior movement from T0 to T2 were less than 2 mm in 89%, less than 1 mm in 72%, and greater than 2 mm in 17% of patients.

**Table 2:** TMJ side-related space dimensions of the BSSO and Control group assessed by CBCT

TMJ space	T0	T1	T1 – T0	P^*^	T2	T2 – T1	P^*^	T2—T0	P^*^
Right side									
Posterior space									
BSSO group (*n* = 36)	2.9 ± 0.8	2.4 ± 0.7	-0.6 ± 1.1	0.004	2.1 ± 0.7	-0.2 ± 0.9	0.165	-0.8 ± 0.9	< 0.001
Control group (*n* = 25)	2.8 ± 0.8	2.2 ± 0.4	-0.5 ± 0.9	0.004	2.4 ± 0.9	0.2 ± 0.9	0.266	-0.3 ± 1.0	0.098
Superior space									
BSSO group (*n* = 36)	3.6 ± 0.1	3.4 ± 0.8	-0.2 ± 1.1^§^	0.263	3.5 ± 0.7	0.1 ± 0.9	0.415	-0.1 ± 0.9	0.556
Control group (*n* = 25)	3.5 ± 0.9	4.1 ± 1.1	0.6 ± 1.6^§^	0.093	3.9 ± 0.7	-0.2 ± 1.5	0.496	-0.3 ± 0.9	0.076
Medial space									
BSSO group (*n* = 36)	2.3 ± 0.7	3.2 ± 0.9	0.9 ± 1.0^§^	< 0.001	2.2 ± 0.7	-1.0 ± 0.8	< 0.001	-0.07 ± 0.7	0.562
Control group (*n* = 25)	2.6 ± 0.7	2.8 ± 0.8	-0.2 ± 0.6^§^	0.136	2.6 ± 0.7	0.2 ± 0.6	0.192	-0.0 ± 0.1	0.435
Left side									
Posterior space									
BSSO group (*n* = 36)	3.0 ± 1.0	2.3 ± 0.7	-0.7 ± 1.2	< 0.001	2.0 ± 0.5	-0.3 ± 0.8	0.026	-1.0 ± 1.0^§^	< 0.001
Control group (*n* = 25)	2.8 ± 1.1	2.4 ± 0.6	-0.3 ± 1.0	0.121	2.6 ± 0.1	0.1 ± 1.1	0.520	-0.2 ± 1.2^§^	0.464
Superior space									
BSSO group (*n* = 36)	3.4 ± 1.0	3.6 ± 0.6	0.2 ± 1.1	0.260	3.1 ± 0.8	-0.5 ± 0.9	< 0.001	-0.4 ± 1.0	0.032
Control group (*n* = 25)	3.6 ± 0.9	4.4 ± 1.4	0.8 ± 1.7	0.024	3.8 ± 0.6	-0.5 ± 1.6	0.104	-0.3 ± 1.0	0.158
Medial space									
BSSO group (*n* = 36)	2.4 ± 0.9	3.5 ± 1.2	1.0 ± 0.9	< 0.001	2.3 ± 0.7	-1.2 ± 1.0	< 0.001	-0.2 ± 0.5	0.051
Control group (*n* = 25)	2.6 ± 0.8	2.8 ± 0.6	0.2 ± 0.5	0.072	2.7 ± 0.8	-0.2 ± 0.5	0.140	0.1 ± 0.1	0.086

*TMJ* Temporomandibular joint, *CBCT* Cone-beam computed tomography, *BSSO* Bilateral sagittal split osteotomy, *T0* Before surgery, *T1* Immediately after surgery, *T2* Mean follow-up at 12.1 ± 2.8 month after surgery, *P*: Probability of type I error

^*^ Paired sample t-test

^*§*^ Significant difference with independent t-test

**Table 3:** Total TMJ space dimensions of the BSSO and control group assessed by CBCT

TMJ space	T0	T1	T1 – T0	*P*	T2	T2 – T1	*P*	T2—T0	*P*
Posterior space									
BSSO group (*n* = 72)	3.0 ± 0.9	2.3 ± 0.7	-0.6 ± 1.2	< 0.001^*^	2.1 ± 0.6	-0.3 ± 0.9	0.011^*^	-0.9 ± 1.0	< 0.001^*^
Control group (*n* = 50)	2.8 ± 0.9	2.3 ± 0.5	-0.4 ± 0.9	0.002^*^	2.5 ± 0.9	0.2 ± 1.0	0.222	-0.3 ± 1.1	0.097
Superior space									
BSSO group (*n* = 72)	3.5 ± 1.0	3.5 ± 0.7	-0.0 ± 1.1^§^	0.961	3.3 ± 0.8	-0.2 ± 1.0	0.058	-0.2 ± 0.9	0.043^*^
Control group (*n* = 50)	3.6 ± 0.9	4.2 ± 1.3	0.7 ± 1.6^§^	0.004^*^	3.9 ± 0.7	-0.4 ± 1.5	0.096	0.3 ± 0.9	0.023^*^
Medial space									
BSSO group (*n* = 72)	2.4 ± 0.8	3.3 ± 1.1	1.0 ± 0.0^§^	< 0.001^*^	2.3 ± 0.7	-1.1 ± 0.9^§^	< 0.001^*^	-0.1 ± 0.6^§^	0.101
Control group (*n* = 50)	2.6 ± 0.8	2.8 ± 0.7	0.2 ± 1.6^§^	0.019^*^	2.6 ± 0.7	-0.2 ± 0.6^§^	0.047^*^	0.3 ± 0.1^§^	0.079

*TMJ* Temporomandibular joint, *CBCT* Cone-beam computed tomography, *BSSO* Bilateral sagittal split osteotomy, *T0* Before surgery, *T1* Immediately after surgery, *T2* Mean follow-up at 12.1 ± 2.8 month after surgery, *P* Probability of type I error

^*^ Significant difference with paired sample t-test

^*§*^ Significant difference with independent t-test

**Fig. 3:**
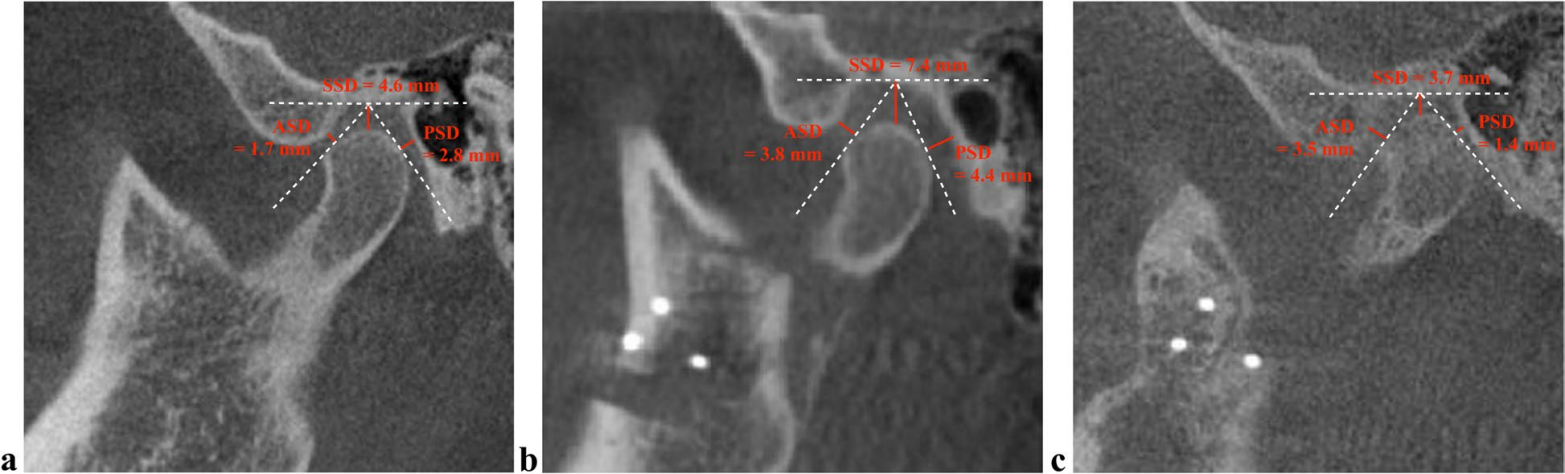
Condylar position changes in a 36-year-old female patient. **a** Condylar location at a concentric position in the glenoid fossa before BSSO surgery. **b** The condyle moved inferiorly immediately after surgery. **c** The condyle changed to a superoposterior position 1 year after surgery

**Fig. 4:**
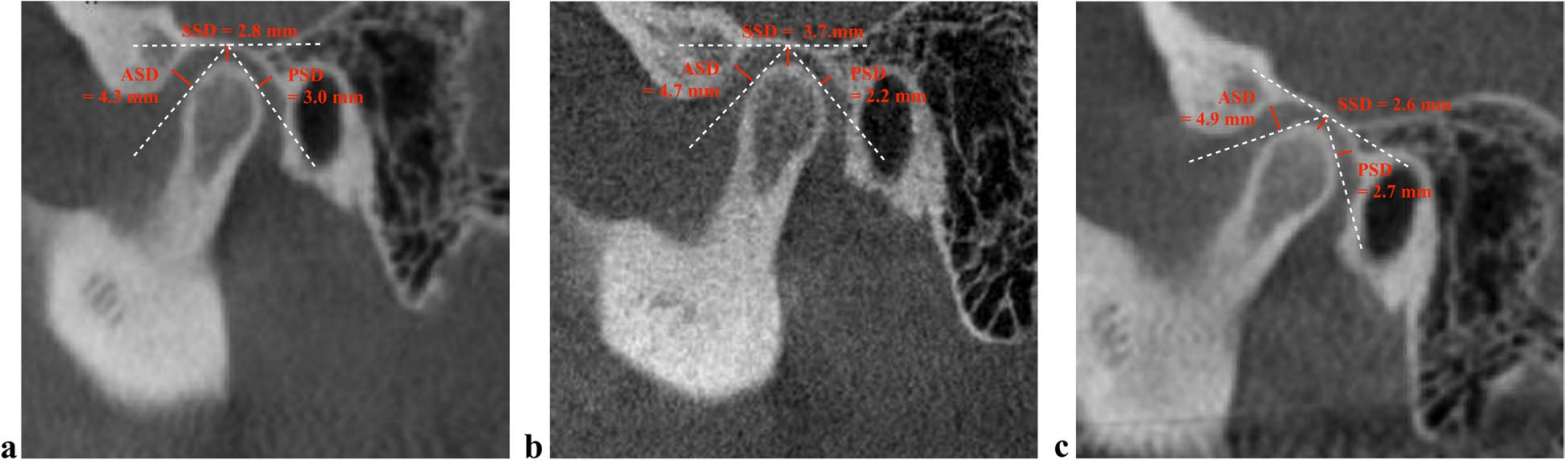
Condylar position changes in a 24-year-old female patient. **a** The condyle was located at a concentric position in the glenoid fossa before cyst removal surgery. **b** The condyle moved posteriorly immediately after surgery. **c** The condyle remained in a posterior position at 1 year after surgery

Most condyles of the BSSO group at T0 were found in an anterior position (44%), whereas at T1 and T2, most condyles were located in a concentric (61%) and posterior position (47%), respectively. In the control group, most condyles were located in a concentric position at T0 (54%), T1 (74%), and T2 (52%). For the BSSO group, the condylar positions (i.e., anterior, concentric, and posterior) revealed a significant association with the time points of investigation (*p* < 0.05) (Fig. 3). For the control group, only the concentric condylar position showed a significant association with the time points of investigation (*p* = 0.046) (Fig. 4) (Table 4).

**Table 4:** TMJ condylar positions of the BSSO and control group assessed by CBCT

**TMJ condylar position**	**T0**	**T1**	**T2**	***P***
Anterior				
BSSO group (*n* = 72)	32 (44%)	9 (13%)	10 (14%)	< 0.000^*#*^
Control group (*n* = 50)	16 (32%)	7 (16%)	12 (24%)	0.103
Concentric				
BSSO group (*n* = 72)	24 (33%)	44 (61%)	28 (39%)	0.002^*#*^
Control group (*n* = 50)	27 (54%)	37 (74%)	26 (52%)	0.046^*#*^
Posterior				
BSSO group (*n* = 72)	16 (22%)	19 (26%)	34 (47%)	0.003^*#*^
Control group (*n* = 50)	7 (14%)	6 (12%)	12 (24%)	0.226

*TMJ* Temporomandibular joint, *CBCT* Cone-beam computed tomography, *BSSO* Bilateral sagittal split osteotomy, *T0* Before surgery, *T1* Immediately after surgery, *T2* Mean follow-up at 12.1 ± 2.8 month after surgery, *P* Probability of type I error

^*#*^ Significant with chi-square analysis

In the GEE statistical analysis, no significant differences were found regarding changes in PSD (β = -0.32, *p* = 0.144), SSD (β = -0.16, *p* = 0.607), and MSD (β = 0.18, *p* = 0.565) between the BSSO and control groups, while the pattern of the decrease over time differed significantly between treatment groups in terms of PSD (β = -0.31, *p* = 0.001), SSD (β = -0.27, *p* = 0.002), and MSD (β = -0.07, *p* = 0.046). The preoperative posterior condylar position showed significant effects on PSD (β = -0.58, *p* < 0.001) and MSD (β = -0.32, *p* = 0.043), while the preoperative central condylar position demonstrated a significant effect on PSD (β = -0.46, *p* < 0.001) The amount of mandibular advancement had no significant effect on changes in PSD (β = -0.02, *p* = 0.474), SSD (β = -0.03, *p* = 0.541), and MSD (β = -0.03, *p* = 0.475) (Tables 5 and 6).

**Table 5:** Multivariate analysis of posterior and superior TMJ space dimensions based on GEE model

Outcome	Parameter	B	SE	95% CI	*P*
Posterior space dimension (mm)	Study group				
	BSSO group (*n* = 72)	-0.32	0.22	-0.74—0.11	0.144
	Control group (*n* = 50)	Reference			
	Mandibular advancement	-0.02	0.03	-0.09—0.04	0.474
	Condylar position				
	Posterior	-0.58	0.11	-0.80—0.36	< 0.001
	Central	-0.46	0.09	-0.65—0.28	< 0.001
	Anterior	Reference			
	Time	-0.12	0.08	-0.28—0.02	0.088
	Time * BSSO group	-0.31	0.10	-0.49—0.12	0.001
Superior space dimension (mm)	Study group				
	BSSO group (*n* = 72)	-0.16	0.32	-0.46—0.78	0.607
	Control group (*n* = 50)	Reference			
	Mandibular advancement	-0.03	0.05	-0.13—0.07	0.541
	Condylar position				
	Posterior	-0.02	0.17	-0.35—0.30	0.902
	Central	-0.08	0.12	-0.31—0.14	0.480
	Anterior	Reference			
	Time	-0.15	0.07	-0.27—0.28	0.017
	Time * BSSO group	-0.27	0.08	-0.43—0.10	0.002

*TMJ* Temporomandibular joint, *GEE* Generalized estimating equation, *BSSO* Bilateral sagittal split osteotomy, *mm* Millimeters, *P* Probability of type I error, *B* Regression coefficient, *SE* Standard error, *ci* Confidence interval

**Table 6:** Multivariate analysis of medial TMJ space dimensions based on GEE model

Outcome	Parameter	B	SE	95% CI	*P*
Medial space dimension (mm)	Study group				
	BSSO group (*n* = 72)	0.18	0.32	-0.44—0.81	0.565
	Control group (*n* = 50)	Reference			
	Mandibular advancement	-0.03	0.05	-0.06—0.13	0.475
	Condylar position				
	Posterior	-0.32	0.16	-0.64—-.0.10	0.043
	Central	-0.17	0.15	-0.46—0.12	0.256
	Anterior	Reference			
	Time	0.01	0.01	-0.01—0.30	0.090
	Time * BSSO group	-0.07	0.04	-0.14—-0.01	0.046

*TMJ* Temporomandibular joint, *GEE* Generalized estimating equation, *BSSO* Bilateral sagittal split osteotomy, *mm* Millimeters, *P* Probability of type I error, *B* Regression coefficient, *SE* Standard error, *CI* Confidence interval

## Discussion

One of the most common complications after BSSO is condylar displacement. Several authors have reported that the condyle may displace in varying directions, thereby modifying the condyle–fossa relationship and inducing postoperative relapse [2–5, 30, 31]. In the present study, statistically significant long-term posterior movements of both condyles were found after BSSO for mandibular advancement, i.e., the condyles moved significantly posteriorly with surgery compared with the initial position before surgery and demonstrated a further posterior movement during the 1-year follow-up. These findings are in agreement with those of other studies [12, 14, 15]. Using CBCT, Carvalho [12] and Chen [15] reported that the condyles move posteriorly with surgery, thereby maintaining this position at the 1-year follow-up. The findings of overall slight posterior movements of the condyles (< 2 mm, 89%; < 1 mm, 72%) of patients are in agreement with those reported by Carvalho et al., who demonstrated that the mean change in condylar position was smaller than 1 mm, while a change of greater than 2 mm was found in only four patients [12]. The results of the present study showed that 17% of the condyles had a condylar displacement of greater than 2 mm at 1-year follow-up. This may be related to manual manipulation of the proximal segment with counterclockwise rotation [32–36] and the development of intra-articular edema at an early postoperative stage [37], while long-term changes may represent the continuation of a physiological adaptation process [9, 38, 39].

For patients in the operative control group, in whom mandibular odontogenic cysts were removed in GA, a significant superior movement of the condyles was observed from T0 to T2 for the total of both sides. Manual manipulation of the mandible during surgery with subsequent intra-articular edema, surface remodeling of osseous TMJ components and altered muscle functional patterns may have induced changes in the positional relationship of the condyle to the glenoid fossa. To the knowledge of the authors, there appears to be no other reference to such a preliminary finding in the current scientific literature. Future studies with larger sample sizes, short- and long-term follow-ups and improved methodologies may be able to provide additional data regarding the effect of OMS procedures on the position and angulation of the condyles and how this can affect bone remodeling and the adaptive capacity of the neuromuscular system.

Several studies have evaluated the relationship between condylar position in the fossa and craniofacial morphology [38–41]. Condyles in Class I were described in a central position of the fossa [39], while condyles in Class II and Division 1 were found to be located more anteriorly than those in Class I or III [41–44]. In the present study, 44% of condyles were positioned anteriorly in the fossa according to the formula of Pullinger and Hollender [29]; this finding agrees with that of Chen et al. [15] who reported 43% of condyles to be located in an anterior position in patients diagnosed with skeletal Class II malocclusion. Compared with the preoperative position, the present study showed that most condyles were displaced in a posterior position (47%) at the 1-year follow-up, while 39% of the condyles were found to be located in a centric position. This finding is inconsistent with that of Chen et al. [15] who found that 28% of condyles were in the posterior position and 65% were in a posterior position at the 1-year follow-up. These observations may be related to the fact that orthognathic surgery may have changed the patients’ occlusion and neuromuscular environment differently, inducing an individual adaptive response from the ligaments and musculature after splint removal. It may be assumed that the centro-posterior position was more stable for condyles in the glenoid fossa after surgery, and this stability was maintained at the 1-year follow-up.

To the best of our knowledge, this is the first study to provide parameters for the estimation of PSD, SSD, and MSD changes of the TMJ in a multivariate design using GEE techniques for analysis. It provides a perspective on the contribution of clinical and CBCT parameters to the occurrence of TMJ space changes. While the clinical parameter of the type of intervention (BSSO vs. control) contributed no significant amount to the change in PSD, SSD and MSD, a clear definition of the PSD and MSD groups was evident for the CBCT variables of baseline posterior and central condylar positions. Therefore, based on this study, the presence of baseline CBCT findings of posterior and central condylar positions may be considered a dominant factor in the definition of PSD and MSD changes following BSSO with GA procedures. Considering the important aspect of postoperative stability, which is the main criterion of successful orthognathic surgery [1, 2], further investigations are necessary to clarify which additional clinical and/or radiological variables may be associated with an elevated risk for pronounced TMJ space changes, while only a prospective cohort study rather than a case‒control study will estimate the etiologic contribution of defined variables to TMJ space changes.

Assessing the risk of developing long-term TMJ space changes should include general, surgical, and biomechanical aspects. Several general factors have the potential to influence the risk of developing changes in TMJ space dimensions. These include age, sex, systemic disease and hormonal factors [45, 46]. Surgical factors comprise the type of surgical intervention, fixation method, and magnitude and direction of surgical change [2], while mechanical factors involve functional overloading, increased friction at the joint, disk displacement, occlusion, and trauma [47–50]. Furthermore, the degree of postoperative biomechanical stress [51] may induce inflammatory changes leading to bone remodeling and progressive condylar resorption [2, 52]. Unlike a case‒control study, a well-controlled cohort study is capable of establishing how specific factors contribute to these changes.

The results of this study may suggest an interaction between GA procedures and the occurrence of changes in postoperative space dimensions of the TMJ. In GA the OMS patient is paralyzed and in a supine position, i.e., operative manual manipulation may potentially produce a forceful posteriorization of the condyle, thereby subsequently inducing short- and long-term pathologic hard and soft tissue changes in the TMJ. In addition, GA itself has been reported to produce condylar movements up to 2 mm posteriorly [53], vertical condylar drops of up to 5.5 mm [54] and inferior–posterior condylar displacements in 63% of subjects [55]. Ongoing research is necessary to determine how well specific CBCT findings of long-term postoperative TMJ space alterations may be linked to GA and/or specific OMS procedures.

The use of CBCT and 3D superimposition has been proposed by several authors for a detailed analysis of condylar position changes in the TMJ [3, 56]. However, these techniques may be associated with errors in the assessment of condylar changes after orthognathic surgery. These have been linked to the slice thickness, window level and width, matrix size, and rendering technique [40, 57]. Furthermore, three-dimensional condylar displacements include changes in position and inclination, i.e., application of the closest surface point method [12] for distance measurements between bone surfaces at the two time points may render difficulties in differentiating true condylar displacement from radiographic error [57].

One potential limitation of this study is that the rotation of the condyle following BSSO was not measured and correlated with the degree of condyle position changes. The role of condylar rotation after mandibular advancement has been discussed controversially. Although some authors described that the use of fixation, including positional screws [13] and miniplates [58, 59] for BSSO provoked inward condylar rotation and medial condylar displacement, others reported that in BSSO for mandibular advancement the fixation with miniplates [16] had no significant effect on the corresponding condylar changes. However, it is known that mandibular advancement usually produces greater condylar displacement if the anterior osteotomy gap is forcefully closed with rigid fixation [60, 61]. In the present study, the lateral flare at the anterior osteotomy site was not maintained by using either a bent titanium plate or bone graft at the anterior gap, i.e., the rigid internal fixation with three bicortical screws may have eliminated the gaps between the proximal and the distal segment, thereby causing a positional change of the condyles. Ongoing research including larger sample sizes and short- and long-term follow-ups should be conducted to provide further data regarding the influence of different fixation techniques on postoperative changes in condylar angulations and how it may influence the development of condylar position changes.

The results of this study indicate that condylar displacement may be a consequence of BSSO with mandibular advancement surgery. Although some of the data showed statistical significance, the potential significance of alterations of the condyle and glenoid fossa morphology, i.e. resorption or remodeling, was not assessed in this study. The occurrence of condylar form alterations after BSSO with mandibular advancement has been assessed in previous systematic reviews [56, 62, 63]. The authors cautioned that while the selected studies showed a small prevalence of condylar form alterations (OR = 0,04), additional high quality prospective research assisted by 3D-imaging technology is needed to allow more definite conclusions [56]. To the best knowledge of the authors, there has been no longitudinal research that compares postoperative changes in condyle and glenoid fossa volumes with those of the condylar position after orthognathic surgery interventions. These aspects clearly demonstrate the necessity of further long-term controlled trials to evaluate not just the condylar position changes but also to quantify and differentiate the volume changes of TMJ structures, specifically the condylar head and the glenoid fossa, following orthognathic surgery [14, 64].

Another important limitation concerns the inadequacy of the control group by including patients with different skeletal malocclusion classes. This limitation raises important aspects and should encourage further investigations that include a homogeneous control group of “skeletal Class II malocclusion" subjects.

Finally, the lack of measuring the changes in the disc position on magnetic resonance imaging (MRI) before and after BSSO warrants further consideration. MRI permits an accurate assessment of the TMJ hard and soft tissue components of the TMJ without any radiation exposure [65]. In a recent study, pre- and postoperative closed- and open-mouth TMJ disc positions were evaluated in skeletal Class II patients who had undergone a BSSO for mandibular advancement. The number of patients with a closed-mouth superior disc position increased from 4 to 13 in 36 patients, reflecting the ability of the disc to assume a closed-mouth superior position in preoperative instances of anterior disc displacement with reduction [66]. Ongoing studies that involve a pre- and postoperative assessment of TMJ disc positions should be encouraged to determine how well specific TMJ disc position changes may influence defined variables of TMJ space changes.

## Conclusion

The data suggest that preoperative posterior condylar position is a significant effect modifier of PSD and MSD over time in this cohort. More research, including more participants undergoing orthognathic and other OMF surgery with GA procedures is needed to draw conclusions on the interaction between baseline parameters and postoperative space dimensions of the TMJ.

## Data Availability

All data generated or analyzed during this study are not publicly available due to ethical and confidentiality reasons Some datasets are available from the corresponding author on reasonable request.

## Abbreviations

ANB: A point-nasion-B-point
ASD: Anterior space dimension
BSSO: Bilateral sagittal split osteotomy
CBCT: Cone-beam computed tomography
DC/TMD: Diagnostic criteria for temporomandibular disorders
GA: General anesthesia
GEE: Generalized estimation equation
MSD: Medial space dimension
OMF: Oral and maxillofacial
PSD: Posterior space dimension
SSD: Superior space dimension
TMJ: Temporomandibular joint
